# Metropolitan Hospital Sunday Fund

**Published:** 1894-08-04

**Authors:** 


### METROPOLITAN HOSPITAL SUNDAY FUND.

### PARTICULARS OF THE AWARDS FOR 1893.

The following is the report of the Committee of Distribution adopted by the Council on the 30th ult., the Lord Mayor in the chair: Your committee have the honour to recommend the payment of awards to 182 institutions, being one more than last year, and an increase of 77 since the first awards were made in 1873. The total amount available for distribution, after allowing for liabilities and the usual current expenses, is £41,800. Of that total £39,865 is now recommended to 127 hospitals and 55 dispensaries. Five per cent. of the total collected, viz., about £2,000, is set apart to purchase surgical appliances (in obedience to Law XII.) in monthly proportions during the ensuing year. The number of deputations representing committees of various hospitals, &c., invited to confer with your committee, and to offer explanations on matters of apparently unsatisfactory character was 20. Of these, one elected to leave the award entirely in the hands of the committee; three took no notice; and, of these three, two were not recommended for any award, and one for a reduced award. Thirteen deputations attended, and of these 13 four are recommended on their full bases, six on somewhat reduced bases, and three are recommended for nil; one application had to be refused as coming from a "penitentiary," and one for declining to comply with the "uniform system of accounts." One hospital has been marked "nil," consequent on the very unsatisfactory condition of its wards and the public complaints made against the professional staff and the general management. Awards are, therefore, recommended to 165 institutions on full bases, and to nine on reduced bases, while to the remaining eight applicants your committee do not feel justified in recommending any awards.

### Twenty-seven General Hospitals.

Charing Cross Hospital, 1,006 5s.; French Hospital, £383 6s. 8d.; German Hospital, £584 11s. 8d.; Great Northern Central Hospital, £335 8s. 4d.; Guy's Hospital, £527 1s. 8d.; Hampstead Hospital, £86 5s.; Italian Hospital, £76 13s. 4d.; King's College Hospital, £1,485 8s. 4d.; London Hospital, £3,545 16s. 8d.; London Homœopathic Hospital, £134 3s. 4d.; London Temperance Hospital, £622 18s. 4d.; Metropolitan Hospital, £584 11s. 8d.; Miller Hospital and Royal Kent Dispensary, £234 15s. 10d.; North-West London Hospital, £345; Poplar Hospital, £306 13s. 4d.; Queen's Jubilee Hospital, nil; Royal Free Hospital, £862 10s.; St. George's Hospital, £1,293 15s.; SS. John and Elizabeth Hospital, £105 8s. 4d.; St. Mary's Hospital, £2,012 10s.; Seamen's Hospital Society, £891 5s.; The Middlesex Hospital, £2,060 8s. 4d.; Training Hospital, Tottenham, £306 13s. 4d.; University College Hospital, £1,293 15s.; West Ham Hospital, £239 11s. 8d.; West London Hospital, £575; Westminster Hospital, £1,054 3s. 4d.

### Special Hospitals.

Five Chest Hospitals.—City of London Hospital for Diseases of the Chest, £939 3s. 4d.; Hospital for Consumption, Brompton, £1,437 10s.; North London Consumption Hospital, £335 8s. 4d.; Royal Hospital for Diseases of the Chest, £335 8s. 4d.; Royal National Hospital for Consumption (Ventnor), £287 10s.

Thirteen Children's Hospitals.—Alexandra Hospital for Hip Disease, £191 13s. 4d.; Barnet Hospital, £38 6s. 8d.; Belgrave Hospital for Children, £153 6s. 8d.; Cheyne Hospital for Incurable Children, £143 14s.; East London Hospital for Children, Shadwell, £479 3s. 4d.; Evelina Hospital for Sick Children, £498 6s. 8d.; Home for Incurable Children, Maida Vale, £67 1s. 8d.; Home for Sick Children, Sydenham, £143 15s.; Hospital for Sick Children, Great Ormond Street, £766 13s. 4d.; North-Eastern Hospital for Children, £287 10s.; Paddington Green Hospital for Children, £153 6s. 8d.; Victoria Hospital for Children, Chelsea, £527 1s. 8d.; "The Vine," Sevenoaks, £23 19s. 2d.

Six Lying-in Hospitals.—British Lying-in Hospital, £47 18s. 4d.; City of London Lying-in Hospital, £95 16s. 8d.; Clapham Maternity Hospital, £15 6s. 8d.; East End Mothers' Home, £38 6s. 8d.; General Lying-in Hospital, Lambeth, £57 10s.; Queen Charlotte's Lying-in Hospital, £316 5s.

Six Hospitals for Women.—Chelsea Hospital for Women, nil.; Hospital for Women, Soho Square, £383 6s. 8d.; Grosvenor Hospital for Women and Children, £95 16s. 8d.; New Hospital for Women, Euston Road, £172 10s.; Royal Hospital for Children and Women, Lambeth, £234 15s. 10d.; Samaritan Free Hospital, Lower Seymour Street, £555 16s 8d.

### Twenty-nine other Special Hospitals.

Cancer Hospital, Brompton, £670 16s. 8d.; London Fever Hospital, £431 5s.; Gorden Hospital for Fistula, Vauxhall Bridge Road, £38 6s. 8d.; St. Mark's Hospital for Fistula, £105 8s. 4d.; National Hospital for the Diseases of the Heart and Paralysis, Soho Square, £95 16s. 8d.; Female Lock Hospital, Harrow Road, £210 16s. 8d.; Male Lock Hospital, Soho, £9 11s. 8d.; Hospital for Epilepsy, Paralysis, and other Diseases of the Nervous System, Regent's Park, £47 18s. 4d.; National Hospital for the Paralysed and Epileptic, £718 15s.; West End Hospital for Diseases of Nervous System, £67 1s. 8d.; British Hospital for Mental Disorders, nil; Central London Ophthalmic Hospital, Gray's Inn Road, £38 6s. 8d.; Royal Eye Hospital, St. George's Circus, £105 8s. 4d.; Royal London Ophthalmic Hospital, £536 13s. 4d.; Royal Westminster Ophthalmic Hospital, £95 16s. 8d.; Western Ophthalmic Hospital, £23 19s. 2d.; City Orthopædic Hospital, £67 1s. 8d.; National Orthopædic Hospital, £43 2s. 6d.; Royal Orthopædic Hospital, £86 5s.; Royal Sea Bathing Infirmary, Margate, £383 6s. 8d.; Hospital for Diseases of the Skin, Stamford Street, £19 3s. 4d.; Western Skin Hospital, nil; St. Peter's Hospital for Stone, £57 10s.; Central London Throat and Ear Hospital, £47 18s. 4d.; Hospital for Diseases of the Throat, Golden Square, £76 13s. 4d.; Royal Ear Hospital, Frith Street, £6 14s. 2d.; Phillip's Memorial Homœopathic Hospital, Bromley, Kent, £33 10s. 10d.; Dental Hospital of London, Leicester Square, £106 7s. 6d.; National Dental Hospital, Great Portland Street, £38 6s. 8d.

### Twenty-three Convalescent Hospitals.

Metropolitan Convalescent Institution, Sackville Street, £622 18s. 4d.; Bexhill Convalescent Home, Sackville Street, £297 1s. 8d.; All Saints' Convalescent Hospital, Eastbourne, £527 1s. 8d.; Mrs. Gladstone's Convalescent Home, Woodford, £115: Hahnemann Convalescent Home, Bournemouth, £33 10s. 10d.; Hanwell Convalescent Home, £19 3s. 4d.: Herbert Convalescent Home, Bournemouth, £33 10s. 10d.; Herne Bay Baldwin-Browne Convalescent Home, £23 19s. 2d.; Homœopathic Convalescent Home, Eastbourne, £9 11s. 8d.; King's College Hospital Convalescent Home, Hemel Hempstead, £95 16s. 8d.; Mrs. Kitto's Convalescent Home, Reigate, £73 13s. 4d.; Mrs. Marshman's London and Brighton Famale Convalescent Home, £76 13s. 4d.; Mary Wardell Convalescent Home for Scarlet Fever, £96 15s. 10d.; Morley House Convalescent Home for Working Men, £182 1s. 8d.; Princess Fredrica's Convalescent Home, East Molesey, £19 3s. 4d.; St. Andrew's Convalescent Home, Clewer, £162 18s. 4d.; St. Andrew's Convalescent Home, Folkestone, £220 8s. 4d.; St. John's Home for Convalescent and Crippled Children, Brighton, £38 6s. 8d.; St. Joseph's Convalescent Home, Bournemouth, £52 14s. 2d.; Convalescent Home for Poor Children, St. Leonard's-on-Sea, £129 7s. 6d.; St. Mary Magdalene's Convalescent Home, Paddington, Nil; St. Michael's Convalescent Home, Westgate-on-Sea, £43 2s. 6d.; Seaside Convalescent Hospital, Seaford, £143 15s.

### Twelve Cottage Hospitals.

Beckenham Cottage Hospital, £47 18s. 4d.; Blackheath and Charlton Cottage Hospital, £67 1s. 8d.; Bromley, Kent, Cottage Hospital, £67 1s. 8d.; Chislehurst, Sidcup, and Cray Valley Cottage Hospital, £47 18s. 4d.; Eltham Cottage Hospital, £23 19s. 2d.; Enfield Cottage Hospital, £38 6s. 8d.: Epsom and Ewell Cottage Hospital, £67 1s. 8d.; Hounslow Cottage Hospital, £38 6s. 8d.; Reigate and Redhill Cottage Hospital, £52 14s. 2d.; Sidcup Cottage Hospital, £28 15s.; Wimbledon Cottage Hospital, £38 6s. 8d.; Woolwich and Plumstead Cottage Hospital, £33 10s. 10d.

### Six Institutions.

Establishment for Gentlewomen, Harley Street, £115; National Sanatorium for Consumption, Bournemouth, £76 13s. 4d.; Invalid Asylum, Stoke Newington, £52 14s. 2d.; Firs Home, Bournemouth, £38 6s. 8d.; St. Catherine Home, Ventnor, £33 10s. 10d.; Royal Mineral Water Hospital, Bath, nil.

### Fifty-five Dispensaries.

Battersea Provident Dispensary, £67 1s. 8d.; Bloomsbury Provident Dispensary, £10 10s. 10d.; Brixton, &c., Dispensary, £57 10s.; Brompton Provident Dispensary, £25 16s. 6d.; Camberwell Provident Dispensary, £92 19s. 2d.; Camden Provident Dispensary, £12 9s. 2d.; Chelsea, Brompton, and Belgrave Dispensary, £45 0s. 10d.: Child's Hill Provident Dispensary, £9 11s. 8d.; City Dispensary, £83 7s. 6d.; City of London and East London Dispensary, £19 3s. 4d.; Clapham General and Provident Dispensary, £36 8s. 4d.; Eastern Dispensary, £38 6s. 8d.: East Dulwich Provident Dispensary, £19 3s. 4d.; Farringdon General Dispensary, £45 2s. 6d.; Finsbury Dispensary, £52 14s. 2d.; Forest Hill Dispensary, £33 10s. 10d.; Gipsy Hill and Upper Norwood Dispensary, £12 9s. 2d.; Hackney Provident Dispensary, £6 14s. 2d.; Hampstead Provident Dispensary, £41 4s. 2d.; Holloway and North Islington Dispensary, £62 5s. 10d.; Islington Dispensary, £43 2s. 6d.; Kensal Town Provident Dispensary, £9 11s. 8d.; Kensington Dispensary, £67 1s. 8d.; Kilburn, Maida Vale, and St. John's Wood Dispensary, £43 2s. 6d.; Kilburn Provident Medical Institution, £27 15s. 10d.; London Dispensary, £19 3s. 4d.; London Medical Mission, Endell Street, £62 5s. 10d.; Margaret Street Infirmary for Consumption, nil; Metropolitan Dispensary, £43 2s. 6d.; Notting Hill Dispensary and Maternity (Provident), £21 1s. 8d.; Paddington Provident Dispensary, £38 6s. 8d.; Pimlico Provident Dispensary (Lupus Street, £17 5s.; Portland Town Dispensary, £19 3s. 4d; Portobello Road Provident Dispensary, £6 14s. 2d.; Public Dispensary, £47 18s. 4d.; Queen Adelaide Dispensary, £43 2s. 6d.; Royal General Dispensary, £41 4s. 2d.; Royal Pimlico Provident Dispensary, £43 2s. 6d.; Royal South London Dispensary, £62 5s. 10d.; St. George's and St. James's Dispensary, £47 18s. 4d.; St. George's Hanover Square Provident Dispensary, £45 0s. 10d.; St. John's Wood and Portland Town Provident Dispensary, £25 17s. 6d.; St. Marylebone General Dispensary, £38 6s. 8d.; St. Pancras and Northern Dispensary, £47 18s. 4d.; South Lambeth, Stockwell, and North Brixton Dispensary, £47 18s. 4d.; South London Medical Aid Institute, nil; Stamford Hill, Stoke Newington, &c., Dispensary, £52 14s. 2d.; Tower Hamlets Dispensary, £43 2s. 6d.; Walworth Provident Dispensary, £11 10s.; Wandsworth Common Provident Dispensary, £9 11s. 8d.; Westbourne Provident Dispensary and Maternity, £14 7s. 6d.; Western Dispensary, £57 10s.; Western General Dispensary, £124 11s. 8d.; Westminster General Dispensary, £23 19s. 2d.; Whitechapel Provident Dispensary, £24 18s. 4d.

